# Incorporating discrete choice experiments into long-term care insurance policy decisions: evidence from China

**DOI:** 10.3389/fpubh.2025.1511001

**Published:** 2025-02-18

**Authors:** Qian Chen, Yuting Dong, Xinyue Lyu

**Affiliations:** ^1^School of Government, Yunnan University, Kunming, China; ^2^School of Humanities and Social Sciences, Guangxi Medical University, Nanning, China

**Keywords:** discrete choice experiments, long-term care insurance, long-term care, health policy, policy decision

## Abstract

**Background:**

Rapid population aging has prompted most emerging economies to consider introducing long-term care insurance (LTCI) as part of a comprehensive social health protection scheme. China is also in the process of establishing its own LTCI framework. However, the details of the scheme are still being explored in pilot cities, and a long-term solution has yet to be finalized. This study aims to examine the insurance preferences of potential enrollees, providing insights to inform further adjustments to the existing framework.

**Methods:**

We examine discrete choice experiment (DCE) evidence from LTCI and evaluate several relevant attributes, including the elimination period, maximum monthly benefit, out-of-pocket rate, and annual premium. The study uses a mixed logit model to elicit respondents’ preferences and willingness to pay (WTP) for these attributes of LTCI and uses physical health status to assess heterogeneity in responses to insurance choice.

**Results:**

We found that most respondents would consider purchasing LTCI, with respondents most preferring the following attributes: (1) an out-of-pocket rate of 25%, (2) a maximum monthly benefit level of 2000 CNY (about 296 USD), and (3) a three-month elimination period. In addition, among the control variables, marital status, personal self-rated health, and the number of children were significant to varying degrees.

**Conclusion:**

The study can provide a reference for further adjustments to the existing scheme, increasing residents’ willingness to participate in insurance and promoting the sustainable development of long-term care insurance.

## 1 Introduction

China’s population is currently getting older. According to China Development Research Foundation (1), the number of individuals in China who are 65 years of age or older is predicted to reach 310 million by 2035, making up 22.3% of the country’s total population, and nearly 380 million by 2050, making up 27.9% of the population. According to UNESCAP (2), 68 million older persons in China are expected to have some form of disability by 2030, with 18.6% of them probably needing help with daily living tasks. Informal family care is no longer adequate to address actual demands as the number of employed women rises and family structures alter (3). Brown (4) describes long-term care insurance as “medical insurance that provides care for an insured person who has lost the basic ability to care for himself or herself because of a permanent physical or mental disability.” By postponing the decline in their daily activities, raising the degree of care required, decreasing the likelihood of hospitalization, and extending their stay at home, long-term care insurance (LTCI) home care services assist the older adult with disabilities live better lives (5–8).

The majority of households cannot afford to pay for long-term care (LTC) (9), which is typically paid for out of pocket in most nations. Due to the narrow market for private long-term care insurance and the high rate of “adverse selection,” these policies are frequently pricey (10). Middle-income nations must make a difficult decision about how to pay for long-term care benefits: through general taxation, social insurance, or private insurance (11). Given the extreme dysfunction of the insurance market on the supply side, private insurance is arguably the least desired choice for the majority of middle-income countries. LTCI markets frequently show limited benefits at premiums that are higher than actuarially reasonable (4). Consequently, social insurance sponsorship is a wise choice. The health and well-being of older adults can be preserved and enhanced by governments assuming responsibility for social preventive and protective measures (12). The proposal to introduce a trial LTCI system in 15 cities was made by the Ministry of Human Resources and Social Security in China in June 2016. 14 more cities were included to the LTCI trial in May 2020 (13). Nevertheless, no long-term plan has been created and the specifics of funding requirements and treatment levels in each test city are still being investigated.

In this study, we propose to gather preferences from randomly selected middle-aged persons in a pilot city for LTCI in China through a discrete choice experiment (DCE) in order to guide future modifications to current programs. Since middle-aged persons are more likely to actively plan for retirement and potential long-term care needs than older folks, we concentrate on them instead of the latter group (14). A research with a Chinese setting would be beneficial to readers worldwide for two reasons. Due to its fast dropping birth rate, China will initially experience a severe aging issue. With the accelerating aging of the population, other countries of the world may soon face this dilemma. Furthermore, China can successfully implement changes and possesses the sociopolitical conditions necessary for social insurance policy reform. Changes in power, interference from other social programs, and opposition from interest groups are all potential threats to policy reform (15). China has the necessary governmental capacity to spearhead the adoption and modification of policies (16).

The choice experiment approach has been used in certain studies to test consumer preferences for long-term care insurance (LTCI) (17–19). However, the majority of these research have focused on commercial LTCI, which has a small insurance market and high premiums; hence, the results are not applicable to the social insurance LTCI reference design. Our study therefore aimed to close this gap by providing answers to two research questions within the framework of a middle-aged representative sample in an LTCI reform pilot city. What are the primary characteristics of LTCI that middle-aged residents of these cities find most appealing? What effects would this social policy change have on long-term care insurance (LTCI) for China and other aging cultures with comparable issues?

## 2 Status of policy promotion and choice of research variables

By integrating insights from comparable studies in the international literature (e.g., (19)) and drawing on China’s policy framework for the implementation of long-term care insurance reform, this study conducts a comprehensive examination of the demand-side and supply-side determinants influencing individual decisions to purchase long-term health insurance.

At the individual level, the decision to purchase insurance is shaped not only by personal risk perceptions and anticipated future needs but also by the structural design and specific attributes of the insurance program itself. Empirical evidence underscores that factors such as income, financial standing, and bequest motives play a significant role in shaping individuals’ decisions to invest in long-term care insurance (20). However, the financial sustainability of long-term care insurance remains a critical issue, as its current funding mechanisms are characterized by instability and uncertainty. Song and Zhu (21) predict that the cost of long-term care insurance in China will exceed 1,000 billion CNY in 2030, reaching 1,293.3 billion CNY, and will reach 3,849.7 billion CNY by 2050. Given the rising cost of premiums in China, raising adequate and consistent funding for long-term care insurance is essential to the program’s sustainable growth. Nevertheless, there are significant challenges facing both the government and private citizens in the particular financing process (22).

Even though national-level guidelines indicate that the health insurance money can be moved to support LTCI, it is dubious in the long run. In essence, the LTCI fund is now an independently financed kind of insurance and has not been split off from health insurance. Regarding the system’s future development, the funding channels and sources allocated from the medical insurance fund are not stable and sustainable. Therefore, the insurance fund formed by citizens’ contributions will be an essential source of social insurance benefits payment (23). Meanwhile, from a personal standpoint, the inhabitants’ income bracket plays a crucial role in defining their purchasing power. According to the National Bureau of Statistics of China (48), China’s urban per capita disposable income in 2023 was 51,800 CNY, or roughly 7,145 US dollars. Under this status quo, the cost of individual contributions is not low, and the financial pressure caused by the cost of care has not been eliminated. In the previous pilot, the pilot cities had diverse standards for annual premium; some municipal financing was based on the pay of the previous year, while others used the per capita disposable income of the residents (24). No single standard was developed. Determining the optimal premium level to balance commercial profitability with consumer price acceptability is essential for fostering the sustainable development of the long-term care insurance market. To address this critical aspect, this study incorporates the annual premium that respondents are willing to pay as a key research variable, reflecting the importance of aligning market dynamics with consumer affordability and preferences.

Secondly, our study of the policy documents reveals that, the maximum monthly insurance benefit, the out-of-pocket rate of the insurance amount, and the elimination period are also mentioned with high frequency in the policy texts, and the differences between pilot cities are quite obvious. The amount of insurance benefits refers to the amount paid by the social security fund in accordance with certain standards and methods when the insured person reaches the state of care agreed in the contract. China Ministry of Human Resources and Social Security’s initial Guidance on Launching the Pilot Long-term Care Insurance System just proposed to “reasonably determine the scope of basic protection and premium standards,” without specifying what constitutes a reasonable determination. The premium differs depending on where you go because of the various funding levels. Different restrictions and premiums are applied to inhabitants who receive different forms of service delivery in certain places, while a uniform quota is used in others. The current daily payment for home care is 100 CNY for the high end and 20 CNY for the low end, with a substantial difference in premium between the two. The daily cost for care at a nursing facility is 105 CNY for the high end and 25 CNY for the low end (25).

The elimination period is a critical provision within the terms and conditions of long-term care insurance, defining the interval between the enrollment date and the time when the insured becomes eligible to receive benefits. For instance, in Chongqing, insured individuals must have contributed to employee medical insurance for at least 24 consecutive months and experienced incapacity for over 6 months before qualifying for long-term care insurance benefits. Given that a significant proportion of incapacitated older adult individuals may pass away within 2 years, the elimination period plays a pivotal role in determining whether these individuals can access long-term care benefits in a timely manner, which is essential for safeguarding their health and well-being. Consequently, this study includes the elimination period as a key research variable to examine its impact on access to care and policy effectiveness.

On the issue of out-of-pocket rate, the latest Guidance on Launching the Pilot Long-term Care Insurance System proposes that “the overall level of fund payments for the costs of eligible care services will be controlled at around 70%” and that localities may make minor adjustments according to their circumstance (26). However, as the reimbursement basis varies from place to place, specific out-of-pocket rate also need to be set scientifically to ensure effective policy implementation.

In summary, this study intends to examine the variables of elimination period, out-of-pocket rate and maximum monthly benefit as selection attributes.

## 3 Methods

### 3.1 Discrete selection tests

The Discrete Choice Experiment (DCE) is a survey-based experiment based on Lancaster’s consumer utility theory. Lancaster (27) argues that a product, service or state can consist of attributes and characteristics, and that respondents make successive choices in a choice set described by the attribute characteristics. The process of respondent selection is the process of weighing the characteristics of each attribute, and its selection reveals its potential utility function. Discrete choice experiment is based on random utility theory and applies a random utility function to reveal respondents’ preferences by introducing random terms to capture unknown potential factors influencing respondents’ choices. Using discrete choice models as the mathematical foundation allows for the quantification of the relative importance or intensity of product, service, or project features. Compared to most other evaluation techniques, discrete choice experiments better simulate real-life situations because the attributes in these experiments are presented in a context that is genuinely reflective of the respondents’ actual lives (28). It is an effective tool for measuring preferences for non-marketed products and has been widely used in the field of health economics and environmental economics research (19, 29, 30). The process of implementing a discrete choice experiment consists of three steps: (i) attribute selection and setting of attribute levels; (ii) choice set design and questionnaire generation; and (iii) data collection.

Based on random utility theory (31), respondents successively make choices that maximize utility in the choice set. Assuming a sample containing I respondents, T choice sets, and J options, the utility $U_{ijt}$ of individual respondent i choosing option j can be expressed as Equation 1:


(1) $$U_{ijt}=X_{ijt}^{\prime }\beta_{i}+\varepsilon_{ijt}$$

$$\left(j=1,2,\ldots ,J\right)$$

$$\left(i=1,2,\ldots ,n\right)$$

$$\left(t=1,2,\ldots ,T\right)$$

where $X_{ijt}^{\prime }$ is the explanatory variable containing the option attributes, and $\varepsilon_{itj}$ is the random error term. For the unobservable stochastic part $\varepsilon_{itj}$ , researchers can only analyze decision-making behavior based on the likelihood or probability of event selection (32). Therefore, researchers have to establish a method to deal with the information related to the random term, i.e., they have to make assumptions about the distribution of $\varepsilon_{itj}$ (33), which has led to the development and evolution of a number of discrete choice models, but each with its own characteristics. Conditional Logit model (CLM) is based on strong assumptions and was widely used in the beginning when dealing with discrete choice data because of its simplicity. Multinomial probit model (MNP) assumes that the random term $\varepsilon_{itj}$ obeys a normal distribution. It breaks through the limitations of CLM, but its computational complexity and the fact that unobservable variables do not obey a normal distribution in many cases make it less widely used (34). Currently, a more commonly used heterogeneity model is the Mixed Logit model (MXL), which breaks through the limitations of the CLM in that the selection probabilities of any DCMs based on the theory of stochastic utility maximization can be estimated by the MXL model. The MXL allows the coefficients of the explanatory variables to be stochastic, with the parameter to be estimated obeying a probability distribution rather than a point estimate, and thus can also be referred to as a Random Parameter Logit model (RPL). MXL allows the parameters to vary randomly across individuals, and by portraying individual heterogeneity through the distribution of the model parameters, the researcher not only captures the heterogeneity of the preferences of the group of respondents, but also allows for correlation between different choice options (35).

The parameter $\beta_{i}$ can be decomposed into two components in the mixed logit model Equation 2.


(2) $$\beta_{i}=b+\omega_{i}$$

$$\left(i=1,2,\ldots ,n\right)$$

*b* is mean of parameter and $\omega_{i}$ is a random term representing the unobserved deviation from the mean b . So the utility function can be written as Equation 3:


(3) $$U_{ijt}=X_{ijt}^{\prime }b+X_{ijt}^{\prime }\omega_{i}+\varepsilon_{ijt}$$

The probability of respondent individual i choosing option j is Equation 4:


(4) $$P_{i}=\overset{T}{\underset{t=1}{\prod }}\frac{\exp\left(X_{ijt}^{\prime }\beta_{i}\right)}{\sum_{j=1}^{J}\exp\left(X_{ijt}^{\prime }\beta_{i}\right)}$$

### 3.2 Choice set and questionnaire design

Respondents compared a range of hypothetical pairs of LTCI strategies described by specific attributes and chose their preferred alternative, including a third option of ‘neither’. The main value of the DCE is that it allows respondents to make a combined trade-off between the attributes and levels of each option (36, 37).

#### 3.2.1 Selection of attributes and levels

The DCE method aims to produce a value measure based on attributes and levels, which allows the total utility to be decomposed into partial utilities for each attribute and level of the AES. The number of attributes in the DCE studies mostly focuses on four to six (38). Based on the discussion in Section 2, we selected the elimination period, out-of-pocket rate, maximum monthly benefit, annual premium as the study attributes, as shown in Table 1.

**Table 1:** Attributes and levels of choice set.

Properties	Definition	Attribute level
Elimination period	How long the disability period is to qualify for benefits.	9 months 6 months 3 months
Out-of-pocket rate	Percentage of out-of-pocket expenses after reimbursement of care costs.	25% 30% 35%
Maximum monthly benefit	Maximum rate of entitlement in the case of severe incapacity and institutional care.	1,000 CNY (about 148 USD) 1,500 CNY (about 222 USD) 2,000 CNY (about 296 USD) 2,500 CNY (about 370 USD)
Annual premium	Annual premiums paid for LTCI.	50 CNY (about 7.4 USD) 100 CNY (about 14.8 USD) 150 CNY (about 22.2 USD) 200 CNY (about 29.6 USD)

#### 3.2.2 Design choice set

When the number of attributes and levels increases, the number of experimental groups increases geometrically, and the number of trials required is enormous. That makes calculations complex and the interpretation of numerous interactions difficult. Therefore, when the number of attributes and levels is large (greater than three), a Fractional Factorial Design (FFD) is generally used to reduce the number of combinations (39) to address the problem of respondents’ decisions being affected by the excessive number of choice sets in the questionnaire. The study used SPSS to obtain eight selection sets after an orthogonal factorial design, an example of which is shown in Table 2. Within each choice set, participants were invited to indicate their most preferred option, option A, option B or option C (“neither”). “Neither” (i.e., option C) essentially represented maintaining the status quo. All option sets were randomly arranged. At the beginning of the questionnaire, the respondents were given basic information about the LTCI policy and the general conditions for obtaining insurance benefits. This background description is presented in layperson’s terms and can be understood even by people with a low level of education.

**Table 2:** Sample choice set for survey.

	Option A	Option B	Option C
Elimination period	3 months	3 months	Neither Option A nor Option B
Out-of-pocket rate	30%	35%	
Maximum monthly benefit	2,500 CNY	2000 CNY	
Annual premium	250 CNY	50 CNY	

#### 3.2.3 Questionnaire design

The questionnaire consisted of four main sections:

- Respondents’ knowledge of long-term care insurance.
- Respondents’ physical status. We presented a comprehensive list of common chronic diseases and common physical activity difficulties in the questionnaire, allowing respondents to select the conditions they suffered from and the activity difficulties they had.
- DCE section. The questionnaire was designed into eight versions, each presenting one of the eight choice sets, with the rest of the questionnaire being consistent.
- Basic characteristics of respondents: including gender, age group, marital status, education level, annual household income, health status, etc.

### 3.3 Study area

Kunming City in Yunnan Province and Nanning City in Guangxi Zhuang Autonomous Region (same level as the province), which were included in the second batch of pilot cities for LTCI, were selected as research sites for this study. The number of permanent residents in Kunming in 2021 is 8.502 million, the annual per capita disposable income is 42,533 CNY (about 6297.85 USD), the total GDP is 722.25 billion CNY (about 106.86 billion USD) and the per capita GDP is about 85,400 CNY (about 12635.44 USD). In 2021, the number of permanent residents in Nanning is 8.75 million, the annual per capita disposable income is 32,679 CNY (about 4837.93 USD), the total GDP is 512.09 billion CNY (about 75.81 billion USD) and the per capita GDP is 58,500 CNY (about 8660.89 USD). These two cities have proposed the following options in their Guidelines for Long-term Care Insurance Pilot. The guidelines details are shown in Table 3.

**Table 3:** Current long-term care insurance policy in the survey area.

	Nanning	Kunming
Annual premium	The current contribution base of the employee medical insurance is used as the contribution base for LTCI. 1) The employee shall share the same proportion of the employer and insured personnel contributions, and their contribution ratio is 0.15%, respectively. 2) Retirees shall pay LTCI premiums according to 0.15% of the contribution rate based on the previous year’s personal pension. 3) The flexibly employed persons and the unemployed persons during the period of receiving unemployment insurance compensation shall pay the premium of LTCI based on the contribution base of the individual participating in the medical insurance for employees in the current period, according to the contribution ratio of 0.30%.	1) The LTCI premium for on-the-job staffs is paid jointly by the employer and the employee at the rate of 0.2% of their medical insurance contribution base. 2) Individuals pay LTCI premiums for flexibly employed persons at a rate of 0.4% of the medical insurance contribution base. 3) For retirees, individual contributions are combined with financial subsidies. The individual contribution is 0.2% of the basis of the retiree’s personal medical insurance account, and the government financial subsidy is 0.2%.
Elimination period	Be incapacitated for more than 6 months.	Be incapacitated for more than 6 months.
Monthly benefit	50% of the average monthly salary of urban employees in 2019 (4,926 CNY) shall be taken as the base for calculating and payment of benefits: If the insured severely disabled person chooses institutional home care services, the Long-term Care Insurance Fund will pay 75% of the monthly care entitlement rate, and the individual will pay the remaining 25%.	For those who choose to receive nursing care services at the designated nursing care service providers, 70% of the monthly nursing care entitlement rate will be paid by the Long-term Care Insurance Fund, and the individual will pay the remaining 30%.
The insured	Who is insured by the basic medical insurance system for urban employees.	Who is insured by the basic medical insurance system for urban employees.

The above scheme is still in its trial period and will be adjusted based on the trial results to form a long-term scheme. Therefore, the study explores whether there are any areas for improvement in the existing schemes by investigating people’s preferences for specific attributes of LTCI.

### 3.4 Data collection and processing

The survey was administered online in October 2022 to Kunming and Nanning citizens aged 40 years or older who conducted the survey, with each questionnaire generating one sample and each sample yielding three rows of data. 500 questionnaires were received for the study, totaling 495 valid questionnaires. Three rows of data were constructed for each choice task for each participant, with one row for each option and an opt-out option, corresponding to 1,485 observations. In each choice profile, we included attribute levels by converting each attribute into a dummy variable. Due to statistical needs, one level of each attribute had to be omitted, with the omitted level serving as the reference group. Each row of data contains a selection dummy variable equal to 1 if the level is selected and 0 otherwise. Statistical results are discussed later.

## 4 Results

### 4.1 Descriptive statistics

Table 4 gives the descriptive statistical analysis results of the entire sample. Respondents were predominantly female, with an average age in their 40s, an average annual household income between 50,000 CNY (about 7413.45 USD) and 100,000 CNY (about 14,826.90 USD), mostly married, with a medium level of education, in good health, and with an average of 2.6 children.

**Table 4:** Descriptive statistics for primary variable.

Variables	Assignment	Mean	SD
Gender	(1) Male = 1; (2) Female = 2	1.503	0.500
Age	(1) 0–40 years = 1; (2) 41–50 years = 2; (3) 51–60 years = 3; (4) 61–70 years = 4	2.319	0.632
Annual household income (CNY)	(1) 0–50,000 = 1; (2) 50–100,000 = 2; (3) 100–200,000 = 3; (4) 200,000 or more = 4	2.236	0.818
Marital status	(1) Unmarried = 0; (2) Married = 1; (3) Divorced or widowed = 2	1.075	0.292
Education level	(1) Primary school = 1; (2) Junior high school = 2; (3) Senior high school/secondary school = 3; (4) Associate degree = 4; (5) Bachelor’s degree = 5; (6) Master’s degree and above = 6	3.206	1.260
Physical health	(1) Very good = 1; (2) Good = 2; (3) Average = 3; (4) Bad = 4; (5) Very bad = 5	2.079	0.872
The number of children	Values based on actual quantities	2.610	0.629

### 4.2 Mix logic model

We use a mixed logit model to relax the assumption that preferences are homogeneous across participants. The parameters are assumed to be randomly distributed and obey a normal distribution. In the model, we used the residents’ preference for LTCI participation as the dependent variable, elimination period, out-of-pocket rate, maximum monthly benefit and annual premium as independent variables, and gender, age group, marital status, education level, annual household income, health status and the number of children as control variables. The results of the model operations are as follows (Table 5).

**Table 5:** Results of mixed logit regression model.

Attributes	Levels	Total observations (1485)	
		Coefficient	SE
Out-of-pocket rate	25%	−0.376	0.256
	30%	−0.286	0.223
	35% (omitted)	-	-
Maximum monthly benefit (CNY)	1,000	−0.202	0.25
	1,500	0.0133	0.297
	2000	0.157	0.215
	2,500 (omitted)	-	-
Elimination period	3 months	1.286***	0.249
	6 months	0.501***	0.17
	9 months (omitted)	-	-
Premium		−0.00223***	0.000746
Gender		−0.204	0.234
Age		0.118	0.227
Edu		0.11	0.0994
Marriage		−0.798*	0.477
Income		−0.076	0.15
Health		0.279*	0.159
Children		−0.475**	0.215
Log Likelihood		−505.80315	
AIC		1059.606	
BIC		1160.516	

*p < 0.1, **p < 0.05, ***p < 0.01.

Based on the model results, it can be seen that respondents most favored the following attributes: (1) a out-of-pocket rate of 25%, (2) a maximum monthly benefit level of 2000 CNY (about 296 USD), and (3) 3 months elimination period. Specifically, the negative mean coefficient for the proportion of out-of-pocket expenses indicates that the higher the proportion of out-of-pocket expenses, the lower the respondents’ willingness to pay. The mean coefficient of maximum monthly benefit is mainly positive, indicating that respondents prefer higher benefit. The positive mean coefficient for the elimination period indicates that the respondents’ willingness to pay decreases with a longer elimination period. In addition, among the control variables, marital status, personal self-rated health, and the number of children were significant to varying degrees. It indicates that respondents who are divorced, in poor health and have fewer children are more likely to pay for LTCI.

We then estimated participants’ marginal willingness to pay (WTP) in each attribute. In the discrete choice experiment, the marginal value of each attribute was calculated to obtain the price respondents were willing to pay to obtain more improvement in the state of the attribute.


$$WTP\;\mathrm{is defined}\;as\;WTP_{non-\mathrm{price}\;\mathrm{attribute}}=-\frac{\beta_{non-\mathrm{price}\;\mathrm{attribute}}}{\beta_{\mathrm{premium}}}$$

The ratios of the coefficients of the different attributes indicate their marginal rates of substitution. Therefore, the respondents’ evaluation of the monetary value of each attribute can be obtained from the ratio of the coefficients of each attribute to the annual premium, and the results are shown in Table 6. The results show that respondents are not concerned about the rate of out-of-pocket expenses and are not willing to pay extra for them. Respondents are willing to pay an additional 97 CNY per year to raise the maximum monthly benefit from 1,000 CNY to 1,500 CNY, and an additional 64 CNY per year to raise the maximum monthly benefit from 1,500 CNY to 2,000 CNY. In addition, when the elimination period is increased from 3 months to 6 months, respondents would be willing to pay 352 CNY /year less.

**Table 6:** Marginal WTP for mixed logit regression results.

Attributes	Levels	WTP
Out-of-pocket rate	25%	−168.610
	30%	−128.251
	35% (omitted)	-
Maximum monthly benefit (CNY)	1,000	−90.583
	1,500	5.964
	2000	70.404
	2,500 (omitted)	-
Elimination period	3 months	576.682
	6 months	224.664
	9 months (omitted)	-

There is often an ‘adverse selection’ in insurance, with people in poor health being more inclined to take out insurance. People with chronic illnesses are at greater risk of disability in old age and are theoretically more likely to take out LTCI. Therefore, we then analyzed the subgroup with chronic illnesses and the results are shown in Table 7. We can see that the model is more significant overall, the absolute values of log Likelihood are smaller, and the values of AIC and BIC are smaller. So this analysis fits the model better than the full sample analysis. Also, the mean coefficients for each attribute are larger, indicating a greater willingness to insure in this subgroup.

**Table 7:** Regression results of subgroup analysis: participants with chronic physical conditions.

Attributes	Levels	Total observations (1095)	
		Coefficient	SE
Out-of-pocket rate	25%	−0.792**	−0.308
	30%	−0.531*	−0.272
	35% (omitted)	-	-
Maximum monthly benefit (CNY)	1,000	−0.43	−0.3
	1,500	0.117	−0.352
	2000	0.144	−0.255
	2,500 (omitted)	-	-
Elimination period	3 months	1.272***	−0.298
	6 months	0.612***	−0.201
	9 months (omitted)	-	-
Premium		−0.00260***	−0.000871
Log Likelihood		−368.04701	
AIC		784.094	
BIC		877.6916	

*p < 0.1, **p < 0.05, ***p < 0.01.

## 5 Discussion

Many emerging nations are now turning their attention to the financing and development of long-term care plans as an integral component of a comprehensive social health protection package, as life expectancy rises and fertility rates drop. More research is necessary to determine people’s preferences for these insurance policies, especially in nations where they are not as widely available. To investigate Chinese preferences for LTCI, we employed the discrete choice experiment method. Despite China’s sizable older adult population, LTCI adoption has not kept up. The maximum chance that someone will buy an insurance policy is represented by the DCE method, which is used to evaluate prospective private demand. Using a mixed logit, we looked at the respondents’ WTP preferences and LTCI characteristics. Our findings suggest that most respondents would at least consider purchasing an LTCI, as only 27% of the total choice set chose the opt-out alternative. We found that respondents most favored the following attributes: (1) a out-of-pocket rate of 25%, (2) a maximum monthly benefit level of 2000 CNY, and (3) 3 months elimination period. Policymakers should carefully consider consumer preferences when designing LTCI programs. However, these designs must strike a delicate balance between the risk tolerance of insurance funds and the pricing of premiums. On the one hand, lower out-of-pocket costs and higher reimbursement rates can enhance the appeal of insurance but may simultaneously increase the financial burden of premiums. On the other hand, extending waiting periods can mitigate the insurer’s payout risks but may negatively impact consumer willingness to purchase coverage. Thus, policymakers and insurers must prioritize the sustainability and affordability of LTCI programs while aligning them with consumer preferences to ensure their long-term viability and social impact.

Among the control variables, the number of children, marital status, and individual self-rated health were significant to varying degrees. Subsequent heterogeneity analyses also suggest that those with chronic conditions are more likely to purchase LTCI. This may be due to the fact that individuals with poor health have a clearer understanding of their future care needs and are therefore more willing to plan ahead and secure their care needs by purchasing LTCI. Some studies have shown that the uncertainty of older people’s care needs and health status has a significant impact on their willingness to purchase LTCI, and those who need daily care or have unstable health status are more inclined to purchase LTCI (40). This ‘adverse selection’ phenomenon is also prevalent in other health insurance policies and may lead to supply-side dysfunctions in the insurance market. To mitigate the adverse effects of this phenomenon on the sustainability of insurance funds, mechanisms such as mandatory enrollment or broader risk-sharing frameworks should be implemented to achieve a balanced distribution of risk. Consequently, developing LTCI within a social insurance framework emerges as a preferred strategy to ensure both equity and financial sustainability.

In addition, persons with fewer children, divorced and widowed persons are also more inclined to purchase long-term care insurance, which may be related to the absence of a family support network and their greater reliance on formal care services to meet future care needs. In general, a high family size indicates an abundance of resources for old age inside the family, which supports the function of family old age (41). There is a replacement effect when children and spouses give informal care. The likelihood that children will assist their older adult parents increases with the number of children (42, 43). Having a spouse who can provide care also lessens the need for official long-term care (44).

The Confucian culture places a strong emphasis on “raising children for old age” and the internal transfer of family wealth across generations as a means of assuming responsibility for old age, with child support serving as the primary means of supporting the family in old life. The cultural conceptualization of old age varies significantly across different contexts, influencing approaches to eldercare. In Europe and the United States, where individualistic values predominate and emphasize the independence and autonomy of older adults, family care constitutes only a small proportion of total care provision. Instead, older individuals often prefer to access care services through professional institutions (45).

In contrast, Japan and South Korea, which share Confucian cultural traditions, prioritize home-based care, with family members serving as the primary caregivers. Confucian values, particularly the emphasis on filial piety and familial responsibilities, shape this caregiving model. Consequently, long-term care insurance policies in these countries often integrate mechanisms such as cash subsidies, care service vouchers, and tax incentives for children who care for older adult family members, providing substantial support for home care (46). In Singapore, while Confucian influences remain prominent, the government has institutionalized filial responsibilities through legal frameworks.

These models exemplifie the integration of Confucian cultural values with modern legal systems and offers a valuable reference point for China as it develops its old-age security policies. When formulating LTCI policies, China should progressively establish a diversified old-age protection system that is family-centered while supplemented by social support, aligning with traditional cultural values of familial caregiving and incorporating lessons from international practices (47). Under the influence of traditional family norms, older adults often prioritize relying on their children for care, potentially suppressing the demand for LTCI. Therefore, the successful implementation of LTCI requires addressing and transforming the societal stigma and moral constraints associated with the perception of “family unfiliality” tied to seeking external caregiving support. Moreover, embedding LTCI within the framework of social insurance can help lower premiums and expand coverage, ensuring broader accessibility for the population. This approach underscores the necessity of establishing a robust social insurance system for LTCI as a critical measure to enhance the well-being and security of older adults.

## 6 Conclusion

This study employs a mixed logit model to analyze Chinese respondents’ preferences for LTCI attributes and their willingness to pay. The results indicate that most respondents exhibit a high level of acceptance toward LTCI, with distinct preferences for key attributes such as out-of-pocket expenses, maximum monthly benefit, and elimination periods. These findings provide valuable guidance for the design of LTCI products, enhancing their market appeal and long-term sustainability.

However, the study is not without limitations. First, the use of choice experiments, which rely on participants’ decisions in hypothetical scenarios, may not fully capture actual behaviors, as respondents’ stated preferences may differ from their real-world purchasing decisions. Additionally, the study’s sample is skewed toward an elite demographic and does not fully represent the broader population. Nevertheless, this sample retains research value, as the initial pilot participants in various regions are predominantly urban employers with relatively high levels of education and income. Looking ahead, future studies should aim to include more diverse respondent groups to better reflect the preferences of the broader beneficiary population, particularly as LTCI policies are expanded and implemented on a wider scale.

## Data Availability

The raw data supporting the conclusions of this article will be made available by the authors, without undue reservation.

## References

[1] China Development Research Foundation. (2020). Available at: https://www.cdrf.org.cn/llhyjcg/5787.htm (Accessed September 01, 2024).

[2] UNESCAP. (2015). Long-term care for older persons in China. SDD-SPPS Project Working papers series: long-term care for older persons in Asia and the Pacific. Available at: https://www.unescap.org/sites/default/files/Long%20Term%20Care%20for%20older%20persons%20in%20China.pdf (Accessed September 01, 2024).

[3] García-Calvente M del Río Lozano M Marcos Marcos J. Desigualdades de género en el deterioro de la salud como consecuencia del cuidado informal en España. Gac Sanit. (2011) 25:100–7. doi: 10.1016/j.gaceta.2011.09.006, PMID: 22088905

[4] Brown JR Finkelstein A. Why is the market for long-term care insurance so small? J Public Econ. (2007) 91:1967–91. doi: 10.1016/j.jpubeco.2007.02.010

[5] Tsutsui T. Implementation process and challenges for the community-based integrated care system in Japan. Int J Integr Care. (2014) 14:e002. doi: 10.5334/ijic.988, PMID: 24478614 PMC3905786

[6] Guo M Chi I Silverstein M. Family as a context: the influence of family composition and family caregiving on long-term care service use in the US and China. The Gerontologist. (2020) 60:588–9. doi: 10.1093/geront/gnaa017, PMID: 38943477

[7] Colombo F Llena-Nozal A Mercier J Tjadens F. Help wanted? Providing and paying for long-term care. Paris: OECD Health Policy Studies (2011).

[8] Newcomer R Harrington C Hulett D Kang T Ko M Bindman A. Health care use before and after entering long-term services and supports. J Appl Gerontol. (2018) 37:26–40. doi: 10.1177/0733464816641393, PMID: 27091879

[9] Costa-Font J Courbage C Swartz K. Financing long-term care: ex ante, ex post, or both? Health Econ. (2015) 24:45–57. doi: 10.1002/hec.3152, PMID: 25760582

[10] Goda G. The impact of state tax subsidies for private long-term care insurance on coverage and Medicaid expenditures. J Public Econ. (2011) 95:744–57. doi: 10.1016/j.jpubeco.2010.11.001

[11] Rhee JC Done N Anderson GF. Considering long-term care insurance for middle-income countries: comparing South Korea with Japan and Germany. Health Policy. (2015) 119:1319–29. doi: 10.1016/j.healthpol.2015.06.001, PMID: 26117093

[12] Sohn M O'Campo P Muntaner C Chung H Choi M. Has the long-term care insurance resolved disparities in mortality for older Koreans? Examination of service type and income level. Soc Sci Med. (2020) 247:112812. doi: 10.1016/j.socscimed.2020.112812, PMID: 32066015

[13] China Development Report. Development trends and policies of China’s population aging. Beijing: China Development Research Foundation (2020).

[14] He J Chou K. Long-term care service needs and planning for the future: a study of middle-aged and older adults in Hong Kong. Ageing Soc. (2019) 39:221–53. doi: 10.1017/S0144686X17000824

[15] Jacobs A Weaver RK. When policies undo themselves: self-undermining feedback as a source of policy change. Governance. (2015) 28:441–57. doi: 10.1111/gove.12101

[16] Wang H. Research progress of the social security policy feedback and its enlightenment to China: a literature review. Soc Policy Res. (2022) 1:65–78. doi: 10.19506/j.cnki.cn10-1428/d.2022.01.004

[17] Akaichi F Costa-Font J Frank R. Uninsured by choice? A choice experiment on long-term care insurance. J Econ Behav Organ. (2019) 7:173. doi: 10.1016/j.jebo.2019.07.012

[18] Nieboer A Koolman X Stolk E. Preferences for long-term care services: willingness to pay estimates derived from a discrete choice experiment. Soc Sci Med. (2010) 70:1317–25. doi: 10.1016/j.socscimed.2009.12.027, PMID: 20167406

[19] He AJ Qian J Chan WS Chou KL. Preferences for private long-term care insurance products in a super-ageing society: a discrete choice experiment in Hong Kong. Soc Sci Med. (2020) 270:113632. doi: 10.1016/j.socscimed.2020.113632 33360249

[20] Allaire B Brown D Wiener J. Who wants long-term care insurance? A stated preference survey of attitudes, beliefs, and characteristics. Inquiry. (2016) 53:1–8. doi: 10.1177/0046958016663728, PMID: 27530238 PMC5812045

[21] Song ZJ Zhu ML. The demand estimation of China’s long-term care insurance and development strategy. Beijing: Tsinghua University Press (2012). p. 43.

[22] Zhao X. Long-term care insurance for the elderly in rural areas: accessibility, problems and countermeasures. J Inner Mongolia Agric Univ. (2021) 23:16–21. doi: 10.16853/j.issn.1009-4458.2021.04.004

[23] Kim H Jung Y Kwon S. Delivery of institutional long-term care under two social insurances: lessons from the Korean experience. Health Policy. (2015) 119:1330–7. doi: 10.1016/j.healthpol.2015.07.009, PMID: 26305121

[24] Wang L Tian Z. Problems of long-term nursing insurance system and countermeasures. Soc Gov Rev. (2021) 4:59–65. doi: 10.16775/j.cnki.10-1285/d.2021.04.012

[25] Zhou L Wang J. Long-term care insurance fund raising and treatment payment policy: based on implementation scheme comparison of 15 pilot cities in China. Res Financ Econ Issues. (2019) 11:89–97. doi: 10.19654/j.cnki.cjwtyj.2019.11.011

[26] Zheng W Yao Y Liu Z Lyu Y. An assessment framework for long-term care insurance system and its application: based on an analysis of three cases. Insur Stud. (2020) 10:65–78. doi: 10.13497/j.cnki.is.2020.10.005

[27] Lancaster KJ. A new approach to consumer theory. J Polit Econ. (1966) 74:132–57. doi: 10.1086/259131

[28] Muhammad FA Wim G Kristof DW. Teachers' preferences for online professional development: evidence from a discrete choice experiment. Teach Teach Educ. (2022) 119:103870. doi: 10.1016/j.tate.2022.103870, PMID: 39906649

[29] Amilon A Kjær A Ladenburg J Siren A. Trust in the publicly financed care system and willingness to pay for long-term care: a discrete choice experiment in Denmark. Soc Sci Med. (2022) 311:115332. doi: 10.1016/j.socscimed.2022.115332, PMID: 36084519

[30] Dias V Belcher K. Value and provision of ecosystem services from prairie wetlands: a choice experiment approach. Ecosyst Serv. (2015) 15:35–44. doi: 10.1016/j.ecoser.2015.07.004

[31] McFadden D. Conditional logit analysis of qualitative choice behavior In: Zarembka P, editor. Frontiers in econometrics. New York: Academic Press (1974)

[32] Louviere J Hensher D Swait J. Stated choice methods: analysis and application. Cambridge: Cambridge University Press (2000).

[33] Hensher D Rose J Greene W. Applied choice analysis: a primer. Cambridge: Cambridge University Press (2005).

[34] Hensher D Greene W. The mixed logit model: the state of practice. Transportation. (2003) 30:133–76. doi: 10.1023/A:1022558715350

[35] McFadden D Train K. Mixed MNL models of discrete response. J Appl Econ. (2000) 15:447–70. doi: 10.1002/1099-1255(200009/10)15:5<447::AID-JAE570>3.0.CO;2-1

[36] De Bekker-Grob EW Ryan M Gerard K. Discrete choice experiments in health economics: a review of the literature. Health Econ. (2012) 21:145–72. doi: 10.1002/hec.1697, PMID: 22223558

[37] Wang N Luo L Pan Y Ni XM. Use of discrete choice experiments to facilitate design of effective environmentally friendly agricultural policies. Environ Dev Sustain. (2019) 21:1543–59. doi: 10.1007/s10668-018-0109-z

[38] Clark M Determann D Petrou S. Discrete choice experiments in health economics: a review of the literature. PharmacoEconomics. (2014) 32:883–902. doi: 10.1007/s40273-014-0170-x 25005924

[39] Ferrini S Scarpa R. Designs with a priori information for nonmarket valuation with choice experiments: a Monte Carlo study. J Environ Econ Manag. (2007) 53:342–63. doi: 10.1016/j.jeem.2006.10.007

[40] He A Qian J Chan W Chou K. Willingness to purchase hypothetical private long-term care insurance plans in a super-ageing society: evidence from Hong Kong. J Aging Soc Policy. (2023) 35:780–805. doi: 10.1080/08959420.2023.2182084, PMID: 36914374

[41] Huang G Guo F Chen G. Utilization of home-/community-based care services: the current experience and the intention for future utilization in urban China. Popul Res Policy Rev. (2023) 42:61. doi: 10.1007/s11113-023-09810-1

[42] Radina M. Mexican American siblings caring for aging parents: processes of caregiver selection/designation. J Comp Fam Stud. (2007) 38:143–68. doi: 10.3138/jcfs.38.1.143, PMID: 39503391

[43] Zweifel P Strüwe W. Long-term care insurance in a two-generation model. J Risk Insur. (1998) 65:13–32. doi: 10.2307/253489, PMID: 38532229

[44] Lakdawalla D Philipson T. The rise in old-age longevity and the market for long-term care. Am Econ Rev. (2002) 92:295–306. doi: 10.1257/000282802760015739 29058393

[45] Norton EC. Long-term care In: Culyer AJ Newhouse JP, editors. Handbook of Health Economics. Amsterdam: Elsevier (2000)

[46] Peng I. The good, the bad and the confusing: the political economy of social care expansion in South Korea. Dev Change. (2011) 42:905–23. doi: 10.1111/j.1467-7660.2011.01724.x, PMID: 22164879

[47] Mu H Chen X. Research on the path and process of changing from rural family supporting to social pensions under the background of aging population in China. Popul Dev. (2015) 21:2–11. doi: 10.3969/j.issn.1674-1668.2015.01.001

[48] China National Bureau of Statistics. (2023). Available at: https://www.stats.gov.cn/xxgk/sjfb/zxfb2020/202401/t20240117_1946643.html (Accessed September 01, 2024).

